# Ventriculoperitoneal shunt fracture in a child

**DOI:** 10.11604/pamj.2021.38.202.28032

**Published:** 2021-02-23

**Authors:** Prastiya Indra Gunawan, Uuk Riuh Kusuma Wardana

**Affiliations:** 1 Department of Child Health, Universitas Airlangga, College of Medicine, Dr Soetomo General Academic Hospital, Surabaya, Indonesia

**Keywords:** Fracture, child, ventriculoperitoneal shunt

## Image in medicine

Ventriculoperitoneal shunt (VPS) fracture is one of malfunction shunt. In pediatric cases, catheter fractures are observed most frequently in the growing period of the patient along with increasing height. Most fractures are reported on the connection side or the side with intense fibrosis because of the repeated pressure. Fracture shunt can be caused by immune reaction, calcification and abrasion leading to shunt dysfunction. A 5-years-old boy presented with headache, vomiting, non-specific abdominal discomfort, high fever, seizure and decrease of consciousness. He was previously hydrocephalic with large head size since birth. A VPS was already inserted twice in 3 months and 5 years old. Neurological examination showed the patient somnolent and Babinski reflexes were positive. Routine hematological investigation revealed leukocytosis. Cerebrospinal fluid (CSF) culture revealed *Staphylococcus aureus* and CSF analysis showed bacterial infection. A contrast-enhanced computed tomography (CT) scan showed left ventriculomegaly and pneumoventricular lateral ventricle, agenesis septum pellucidum, left subdural hygroma in regio fronto-parieto-occipito temporalis. From Plain abdominal X-ray showed of fractured VP shunt tip in abdominal cavity. Emergency surgery was performed to evacuate VPS and antibiotic given based on CSF culture result. The patient was hospitalized about one month and improved leaving neurological sequalae.

**Figure 1:**
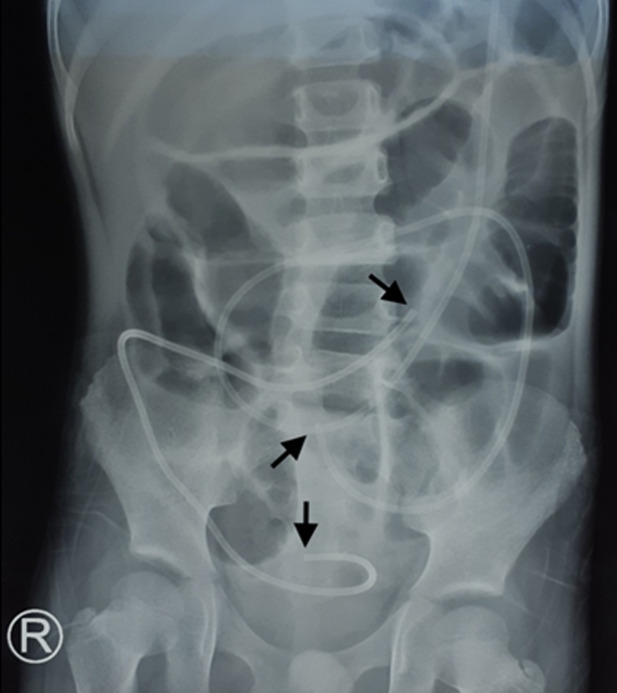
plain abdominal X-ray showed of fractured ventriculoperitoneal shunt tip in abdominal cavity

